# Death Following a Skiing Incident: Severe Exsanguination Due to Rupture of the Left Renal Artery

**DOI:** 10.7759/cureus.57575

**Published:** 2024-04-04

**Authors:** Biliana Mileva, Metodi Goshev, Tihomir Dikov, Mihaela Georgieva, Martina Valcheva, Ivan I Tsranchev, Alexandar Alexandrov, Vesela Ivanova

**Affiliations:** 1 Department of Forensic Medicine and Deontology, Medical University of Sofia, Sofia, BGR; 2 Department of General and Clinical Pathology, University Hospital "Alexandrovska" Medical University of Sofia, Sofia, BGR; 3 Department of Forensic Medicine and Deontology, Medical University of Plovdiv, Plovdiv, BGR

**Keywords:** trauma, recreational sports, mechanism of injury, blunt force trauma, winter sports, abdominal trauma, fatal incidents, autopsy, pattern of injury, snow-skiing

## Abstract

With the growing popularity of winter sports, it is necessary to pay more attention to the types of traumatic injuries that a person can sustain in various incidents related to their practice. We present a case in which an adult man died as a result of a collision with a tree while skiing. Although the deaths are associated with different types and severity of craniocerebral injuries in the majority of the cases, here we are dealing with an abdominal injury with rupture of the stomach, pancreas, and left renal artery. The exact localization of the resulting traumatic injuries and the mechanism of their occurrence were examined. Both macroscopic autopsy findings (gross pathology) and histologically proven ones are presented and described. Presenting this case, we want to raise awareness of the different types of injuries received while skiing, as well as to emphasize the possibility of death in the absence of visible external injuries over the victim's body.

## Introduction

Skiing and snowboarding are some of the most popular piste-based recreational and competitive winter sports, which are gaining more and more popularity worldwide every year [1]. Thousands of years ago, alpine skiing was used as a method of transportation in Scandinavia [2]. The popularity of the sport nowadays results in increasing numbers of reported traumatic injuries associated with falls, collisions, and accidents in the skiing areas [3]. According to McBeeth et al., the most common mechanism of injury is related to falls (while riding off a chair lift), collisions with natural objects or another person, or injury sustained by an avalanche [4]. Such accidents might result in different types of injuries - from superficial ones to severe traumas with possible lethal outcomes. First, knowing the most common type of injury and the mortality risk associated with various kinds of winter sports is essential to knowing the actual risk associated with practicing one sport. Secondly, it could be used to build or develop effective preventive measures [5].

The current case was presented as a poster presentation in the 14th Annual Scientific Meeting of the Balkan Academy of Forensic Sciences, which took place in Istanbul, Turkey, on 5-8 October 2023.

## Case presentation

**Figure 1:**
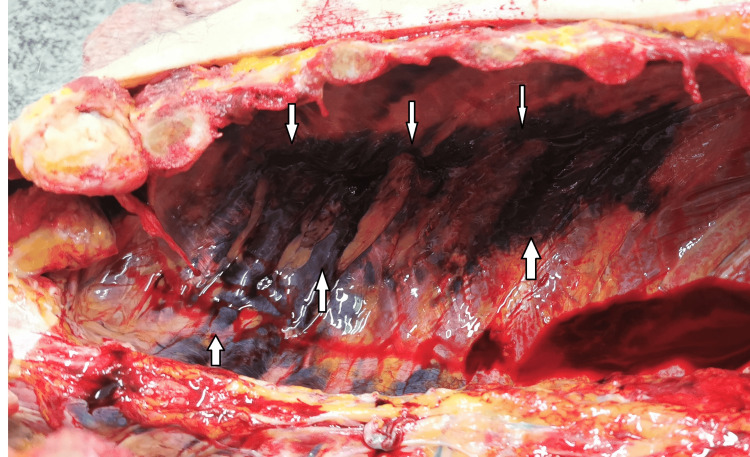
Non-displaced rib fractures. Massive bruises of the soft tissues (white arrows) with intact integrity of the parietal pleura.

A man in his 70s died in a skiing incident following a collision with a tree. The deceased's body was sent for an autopsy, which was performed the next day. The external examination of the body did not show any significant findings, apart from a small superficial abrasion situated at the abdominal area, left to the midline, measuring approximately 2.5/2 cm. The lividities were scanty, located at the posterior, non-pressed areas of the body. The internal examination revealed the following: bilateral non-displaced rib fractures - from second to eighth ribs on the left were fractured on the mid axillar anatomical line, and from the right, fourth to sixth ribs were fractured with a line of fracture having an oblique direction from mid axillar to front axillar anatomical line. The parietal pleura of the thoracic cavity was intact. The soft tissues were massively bruised but not lacerated. There was no blood in the thoracic cavity (Figure 1).

**Figure 2:**
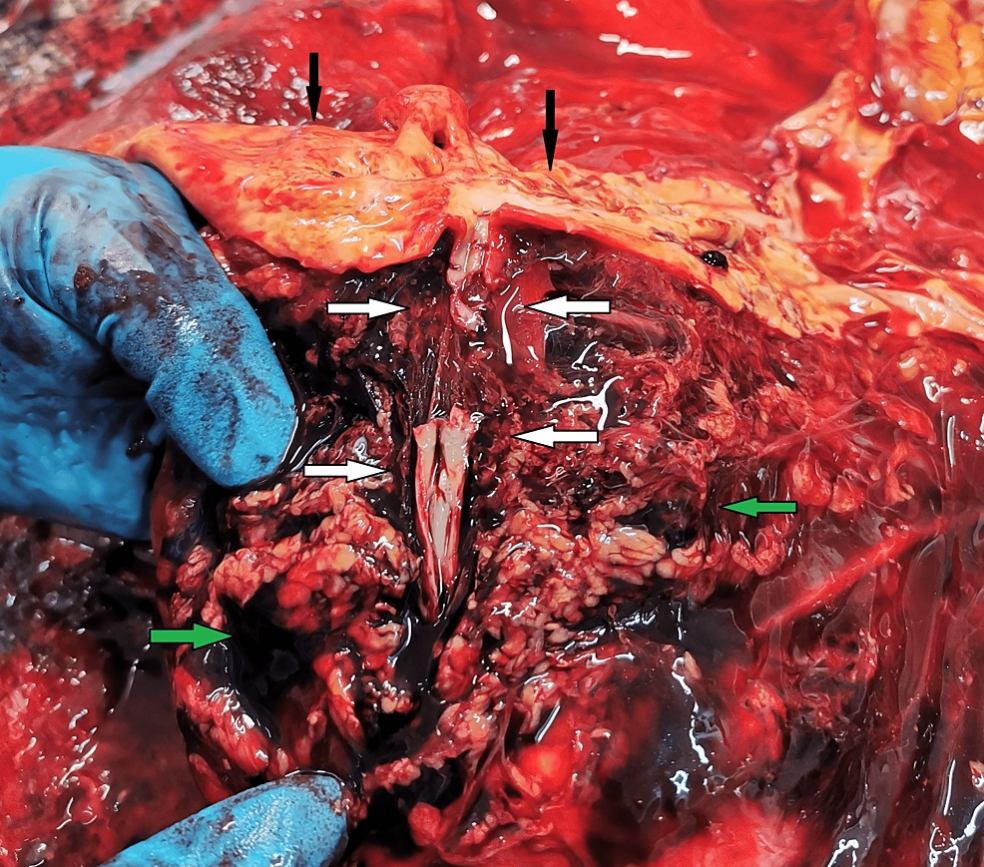
Traumatic rupture of the left renal artery (white arrow); abdominal aorta with atherosclerotic plaques (black arrow); massive bruise of the surrounding soft tissues (green arrow).

**Figure 3:**
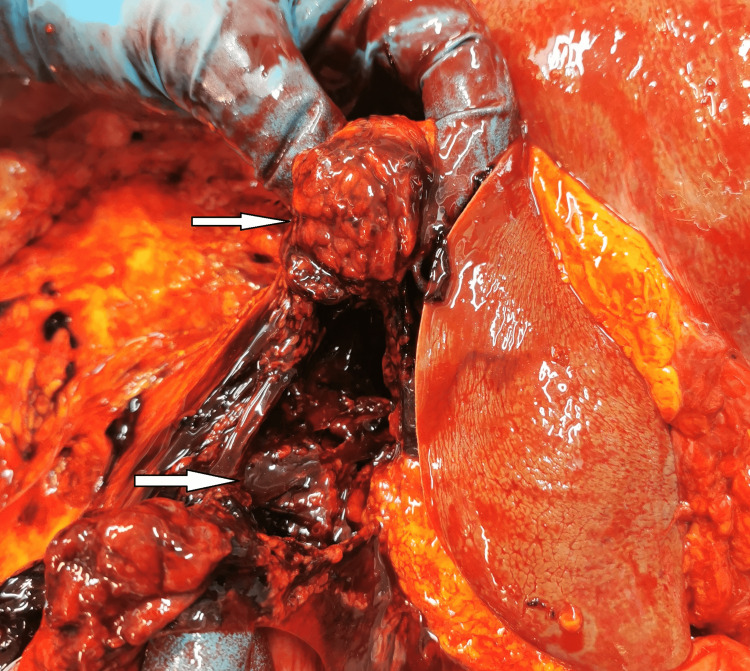
Complete separation of the pancreas.

**Figure 4:**
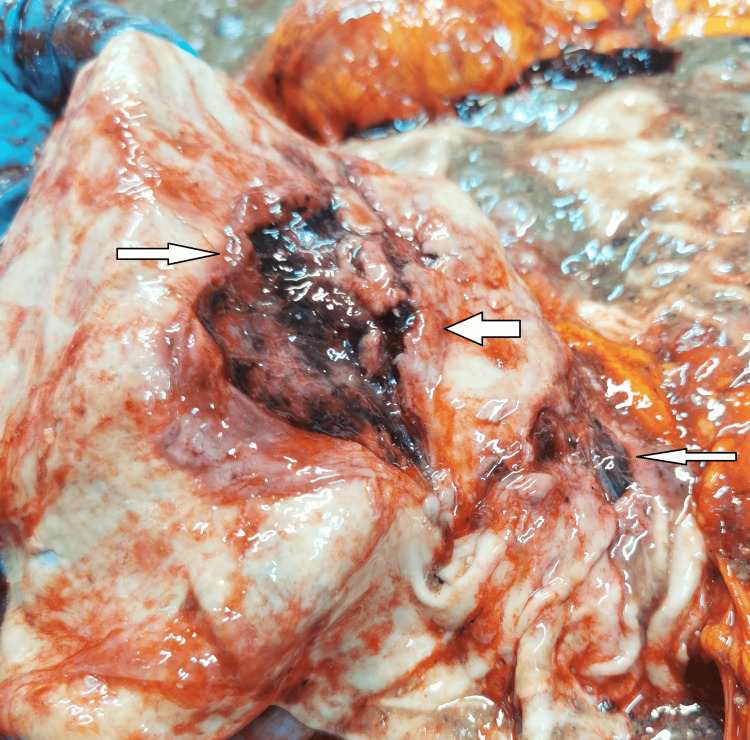
Irregularly shaped tears of the internal layer of the stomach mucosa.

The lungs had massive areas of contusions, more extensive on the left. There were no lacerations of the lungs. The abdominal cavity and retroperitoneal compartment were full of dark liquid blood, measuring approximately 1.5-2 liters. A careful examination of the organs situated in the abdominal cavity revealed the following findings: tearing of the left renal artery (Figure 2), complete separation of the pancreas (Figure 3), and contusion with two irregularly shaped tears of the internal layers of the stomach mucosa (Figure 4).

**Figure 5:**
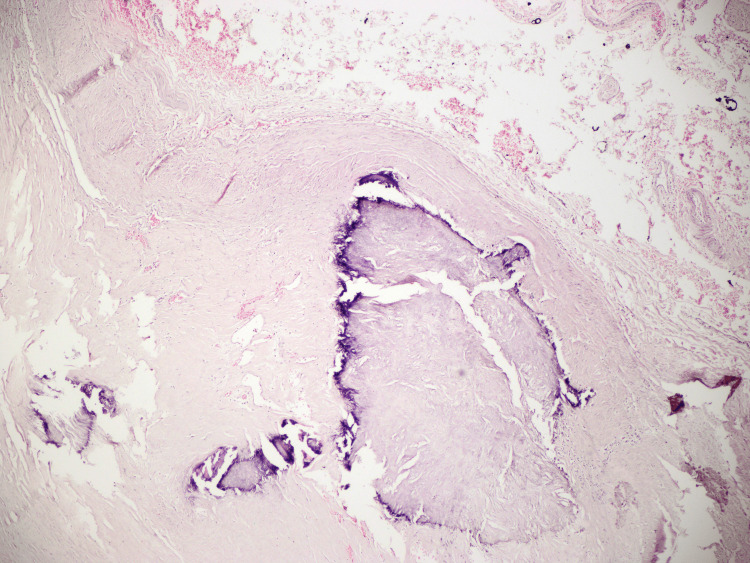
Vascular wall: an artery of elastic-muscular type with atherosclerotic plaques in different stages of development, including calcium deposition.

**Figure 6:**
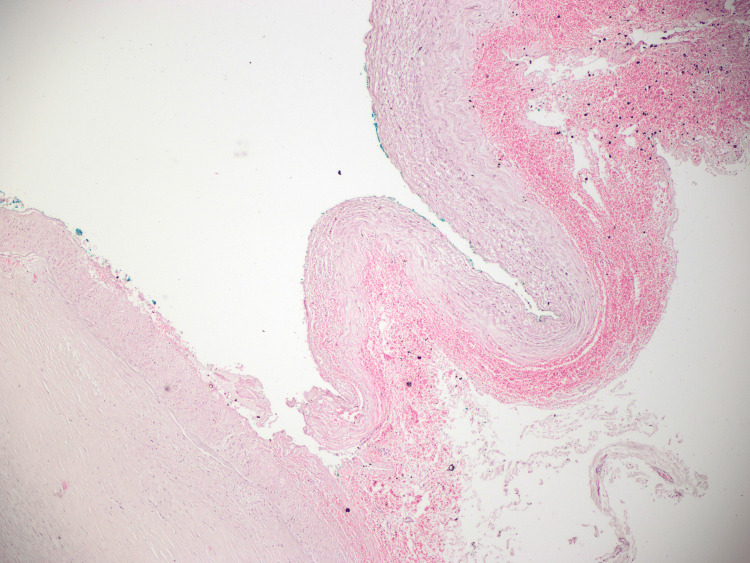
Loss of integrity of the vascular wall with the presence of oblique blood-filled false lumen (marked with green dye), intramural hematoma formation in the outer 1/3 of the vessel wall, and fresh hemorrhages in the surrounding soft tissues.

**Figure 7:**
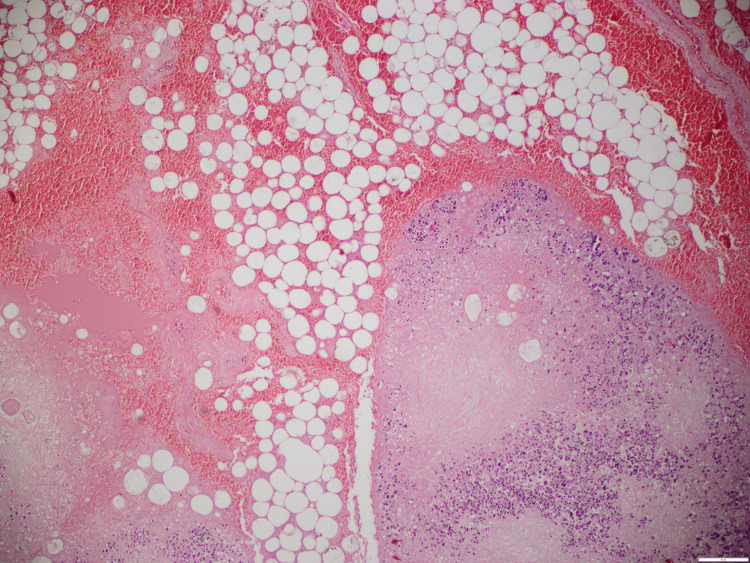
Pancreas: Fresh hemorrhages, lipomatosis, and autolysis.

**Figure 8:**
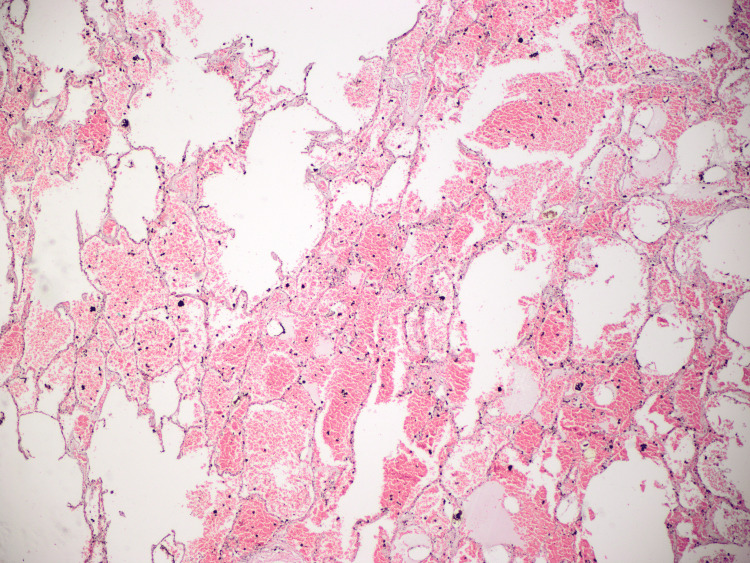
Lung: contusions focus on fresh hemorrhages.

The toxicology result was negative for alcohol and drugs. Tissue samples were taken for further histopathological evaluation and stained with hematoxylin and eosin. The results from the microscopic examination of the tissues confirmed the gross autopsy findings, and are shown and described in detail in the following figures (Figures 5-8).

The cause of death was attributed to exsanguination from the ruptured renal artery and the pancreas.

## Discussion

Skiing and snowboarding are among the most popular winter sports with increasing popularity worldwide, leading to augmentation of the risk for a wide range of injuries [4]. Depending on the situation, deaths occurring while practicing different types of winter sports might be classified into three broad groups - those with traumatic origin, those as a result of sudden cardiac death, or deaths of hypothermia [6]. In their study, Posch et al. analyzed the incidences of fatalities on Austrian ski slopes for 10 years, and the results showed that between the winter seasons 2008/09 and 2017/18, a total of 36 fatalities occurred. In total, 19 were with traumatic origin and 17 were nontraumatic deaths [7]. The leading nontraumatic death was reported to be associated with cardiac system - sudden cardiac death [4,8,9]. Sherry et al., in their study concerning nontraumatic deaths while skiing, reported that of the 15 cardiovascular-related deaths, 13 died from complications related to coronary-artery disease, one case was ruptured aortic aneurysm, and one from cardiac arrhythmia [6].

As previously mentioned, the most common mechanism of traumatic injuries while skiing or snowboarding is related to falls, collisions with natural objects or another person, or sustained by an avalanche [2,4]. A less frequent mechanism of death is the so-called non-avalanche-related snow immersion death known as “tree well death.” The skiers/snowboarders fall into a hidden pit underneath a tree in this situation. In the absence of witnesses, the person will stay trapped below the snow and will die from hypothermia or asphyxiation from the snow falling in [2,10,11]. Most reported traumas are isolated orthopedic injuries that might not require hospital admission [4]. McBeth et al., in their study, made detailed analyses of the injury distribution, which showed that the most injured were the brain, followed by the chest, and different types of fractures - spine fractures or fractures to the extremities. Abdominal injuries are in fifth place, followed by facial, neck, spinal cord, and skull fractures, and hypothermia [4]. As reported by Bianchi et al., for the period between 2008 and 2012, 19 people, on average, died while skiing or snowboarding [1]. Federiuk et al., in their study, report a total of four deaths while practicing winter sports for seven years - the cause of death in three of them was associated with severe head injury, and one was associated with head and thoracic injuries [12]. McBeth et al. report only five people died as a result of their injuries for 10 years, with the leading cause of death associated with fatal brain trauma [4]. Morrow et al. report a total of 16 fatalities during seven ski seasons. In 14 of them, the cause of death was associated with blunt trauma to the head; the remaining two cases were associated with chest and abdominal injuries [13]. Ruedl et al. report in their study a total of 207 registered deaths for five years, 97 of which were of traumatic origin. In 45 of the cases, the head injury was the primary cause of death [14].

As seen from the studies mentioned above, head injuries are the most common severe traumatic injuries associated with such type of winter activity and with the highest fatality prevalence, including different types of skull fractures (mainly fractures of the skull base) and traumatic brain injury (including intracerebral, subarachnoid, and subdural hemorrhage) [2-4,12,15]. Chest trauma is reported to be the second most frequent injury for both survivors and deceased, comprising rib fractures, pneumothorax, and hemothorax [4]. Another frequently observed type of injury is the so-called “tobogganing injury,” which is characterized by falling in a sitting position and, as a result, landing on the buttocks with varying degrees of spinal injuries [15]. In the literature, there is a highly reported incidence of splenic injuries among snowboarders (“Boarder belly") [16].

**Figure 9:**
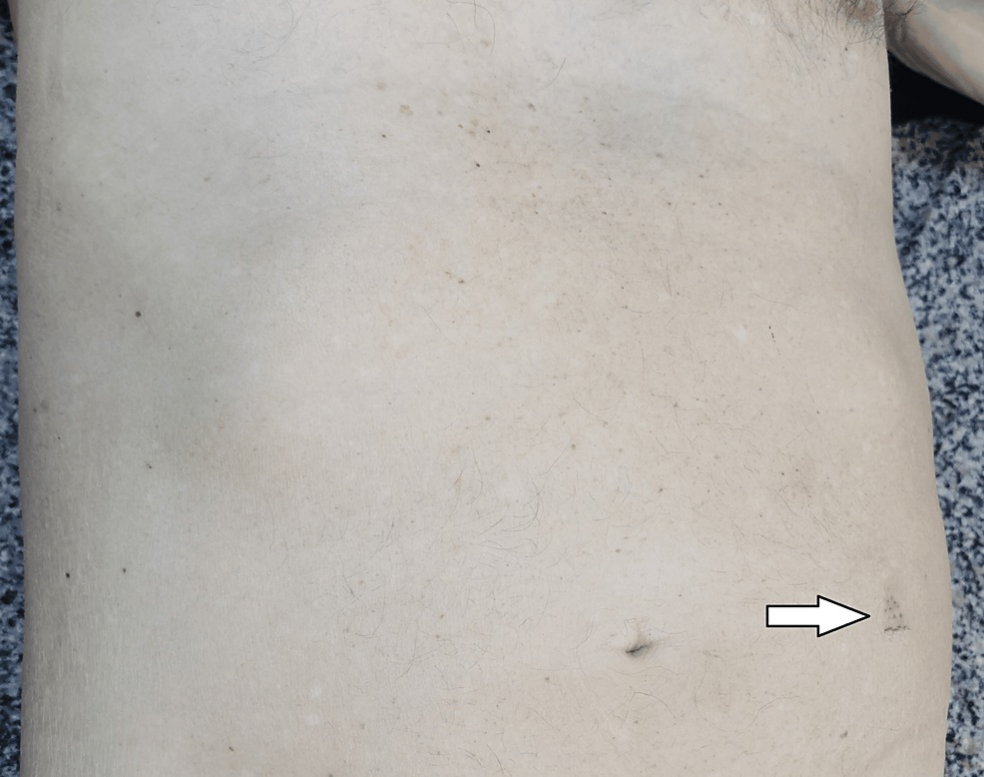
The superficial abrasion in the abdominal area - the only visible external injury (pointed by the white arrow).

We present a rare case in the forensic medical practice of a tree-collision fatality with a severe abdominal injury, which was concluded to be the cause of death. It refers to a severe blunt force trauma - frontal collision with а tree (with the left-anterior surface of the body hitting the object). The last was supported by the presence of а small abrasion in the abdominal area (left side) (Figure 9), as well as by the presence of more severe types of injuries on the left side of the body.

The location of the lethal traumatic injuries (stomach, pancreas, and renal artery), relatively in front of each other, led to the conclusion that during the impact, this particular area was probably hit by a protruding element of the tree (bump, branch, or other similar) which injured the internal organs because the abdominal area is movable, not protected by bones, as is the thorax by the ribs. In the genesis of traumatic injuries, a crucial role also plays the sudden acceleration-deceleration of the body's biological structures, leading to additional traumatic changes - contusions of the internal organs.

Last but not least, we want to raise awareness among doctors involved in rescuing and providing medical assistance to victims of various incidents, emphasizing the fact that in some cases, the victim may not have visible external traumatic changes and injuries but may have severe and even fatal internal injuries, as is the situation in the case presented. Each patient must receive a special and thorough examination, with or without visible external traumas, to avoid misdiagnosed or missed internal injuries, which may have fatal outcomes.

Although death is a rare event in such winter sports, it is crucial to raise awareness of the possible fatal outcomes, which could change snow riders’ behavior and reduce the number of incidents. Studying the types of sustained injuries and their localization is essential for medical practitioners. Knowing what to expect could help them make different strategies for fast and adequate medical treatment when needed.

## Conclusions

The current case raises awareness of the possible fatal outcomes when practicing winter sports. It presents the type of sustained injuries and the cause of death following a collision with a tree - severe blood loss following a rupture of the left renal artery.
